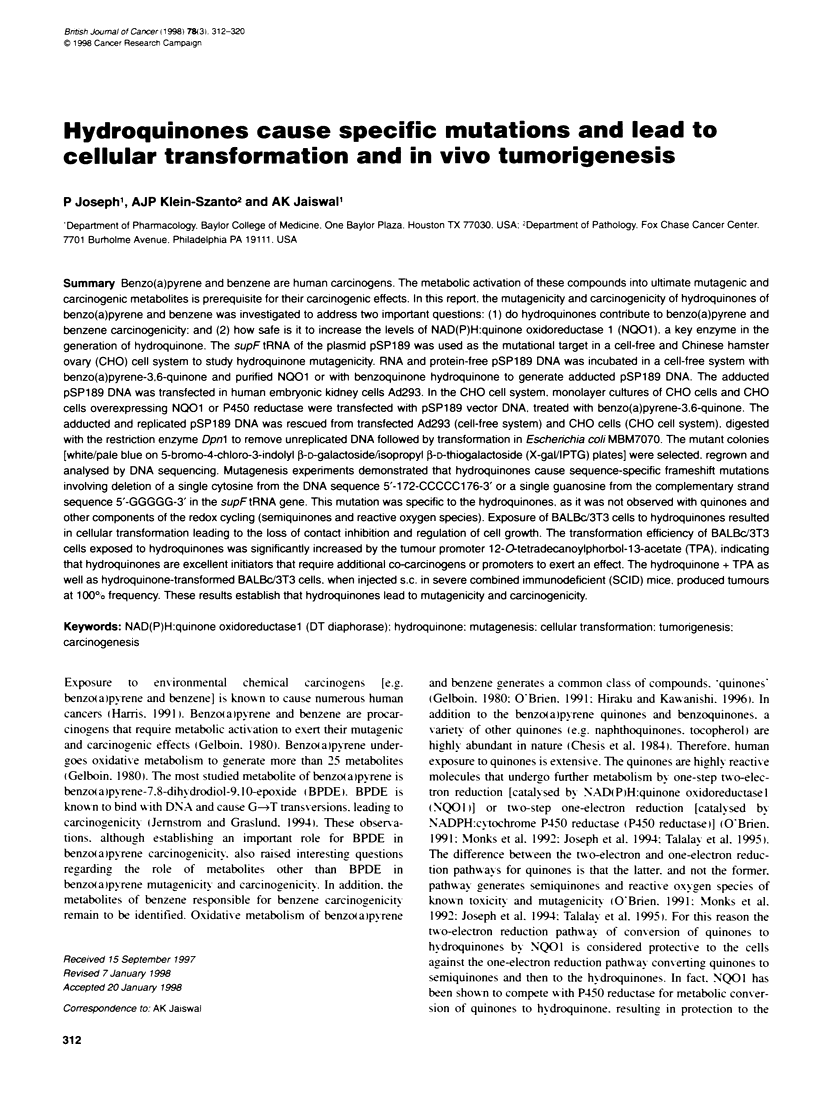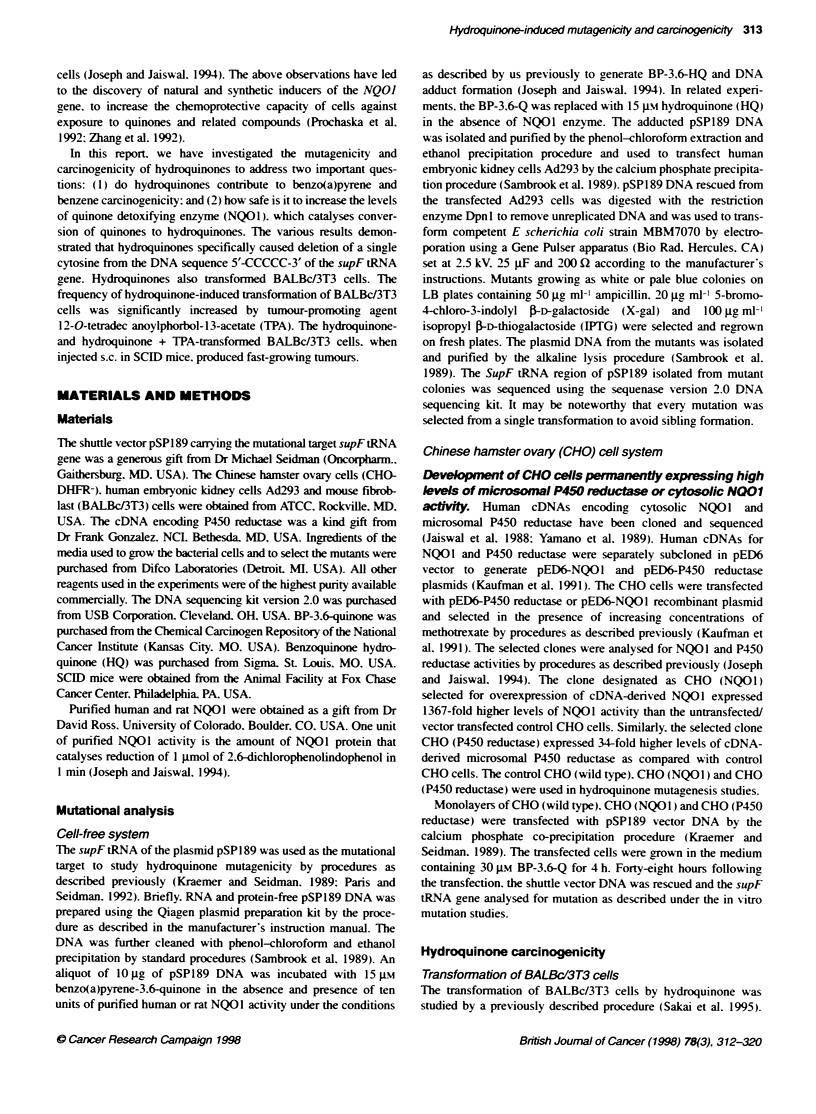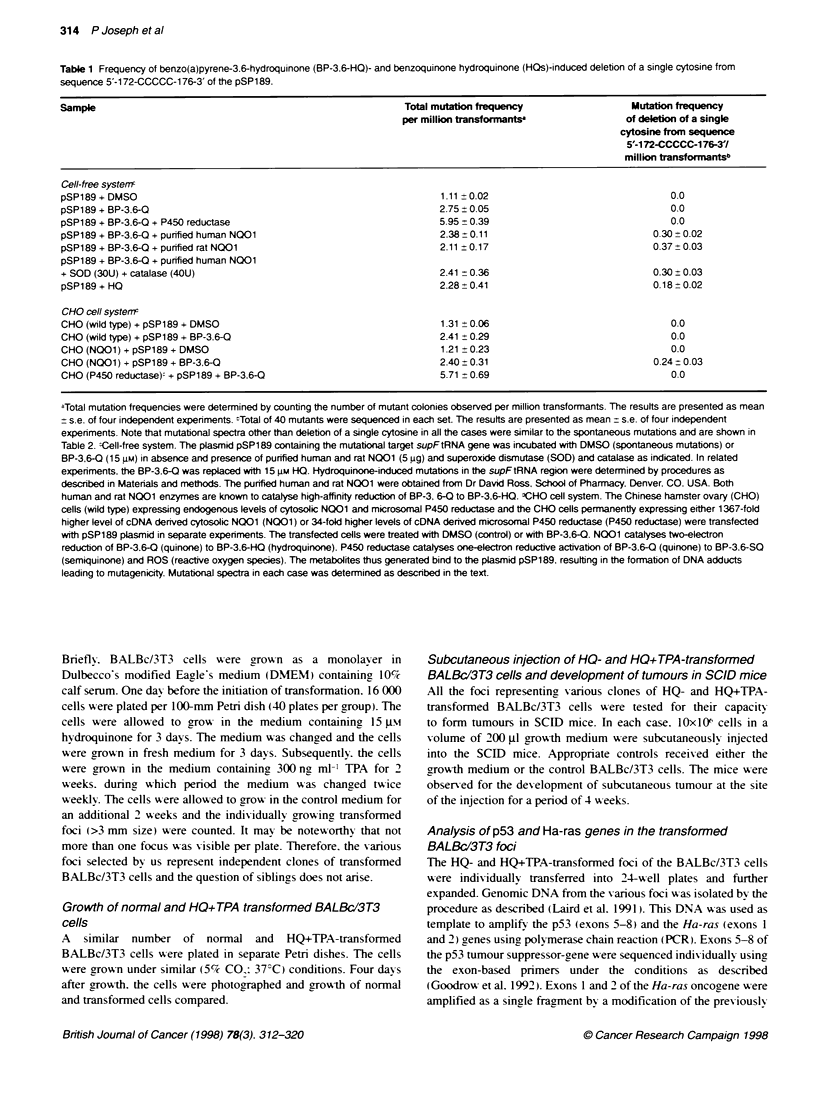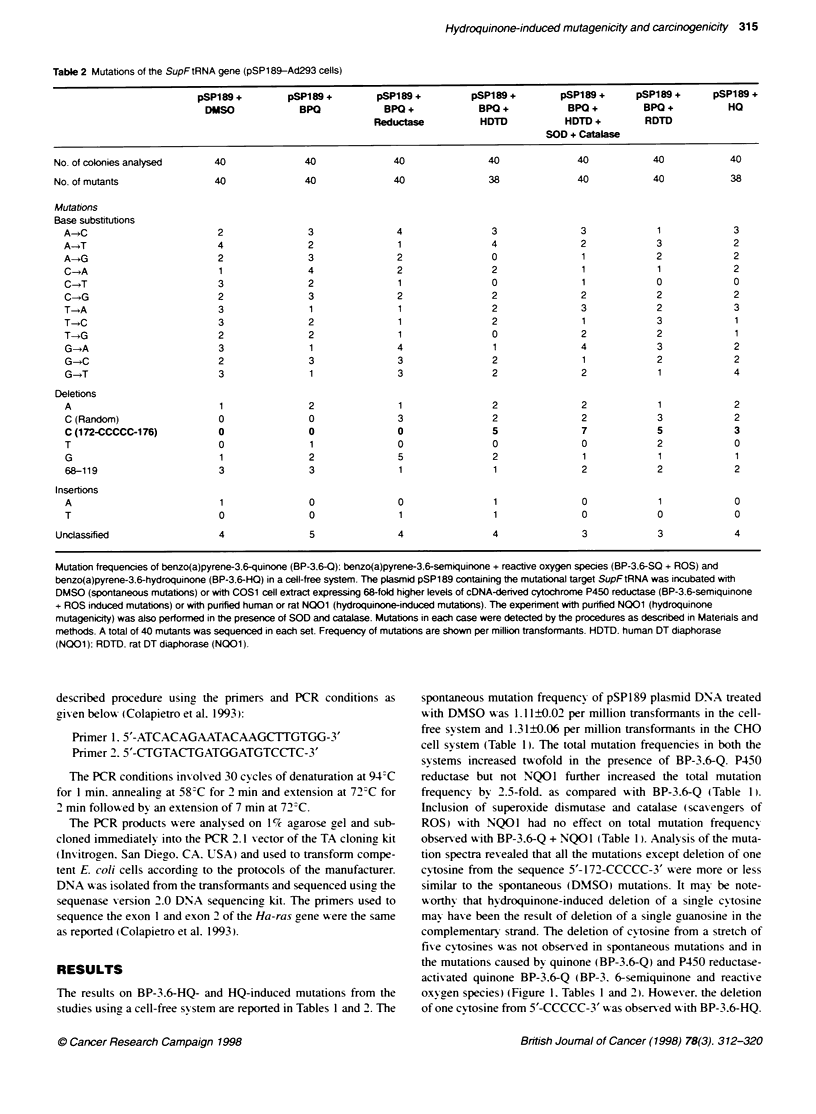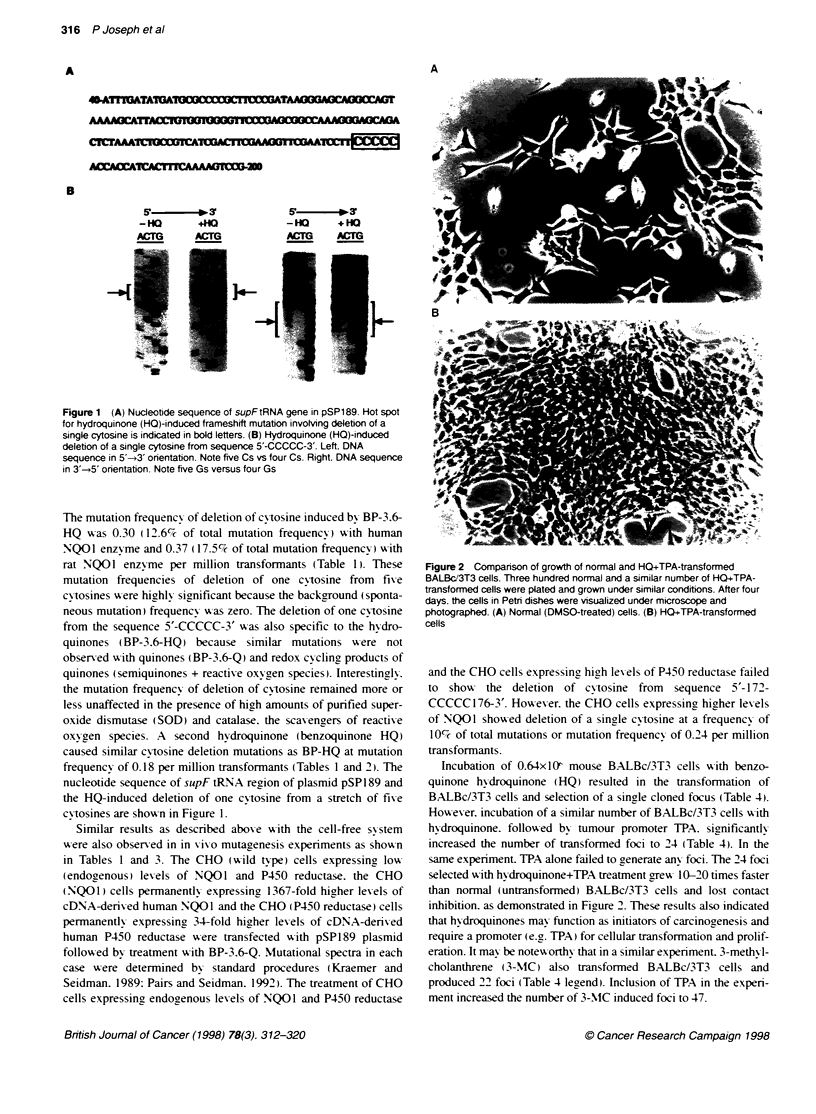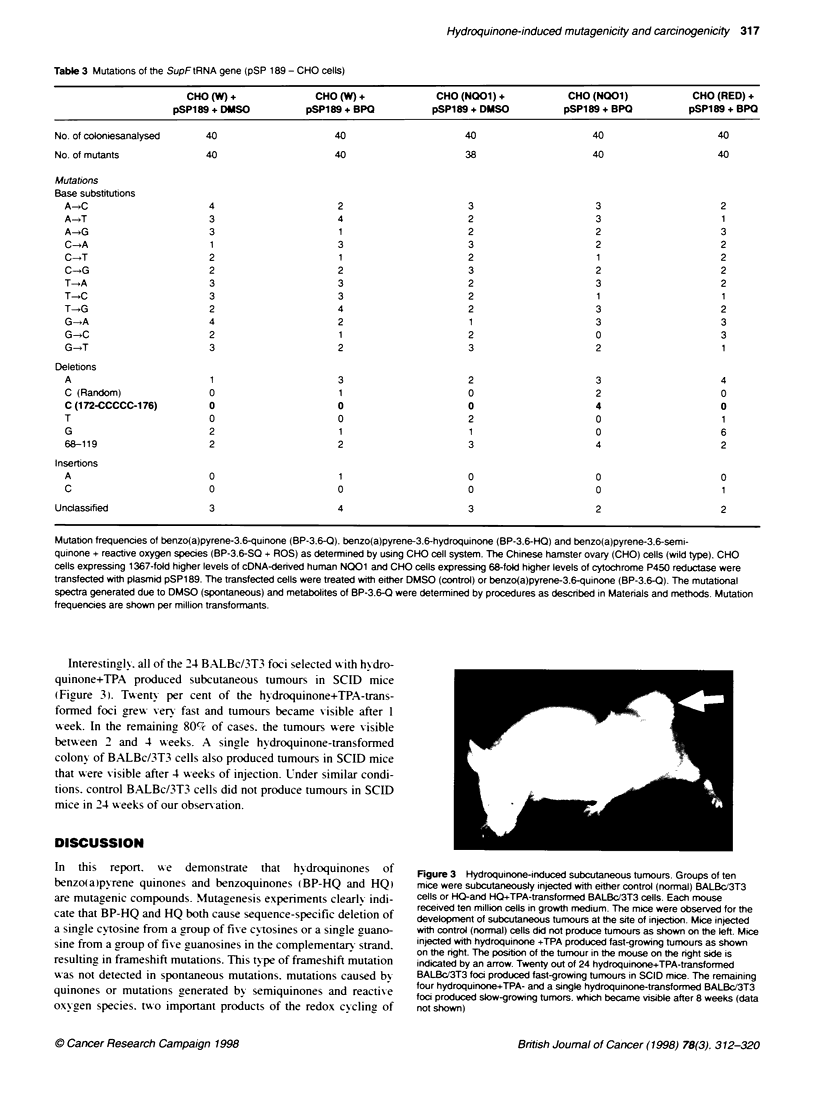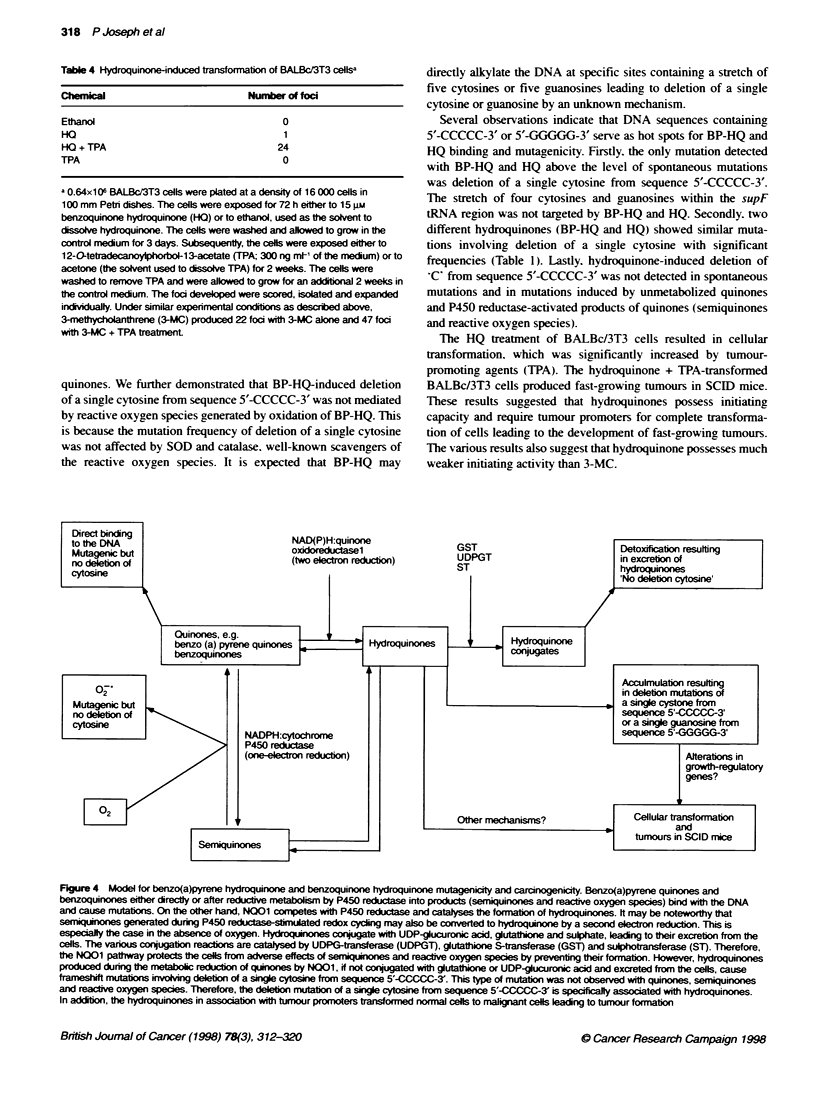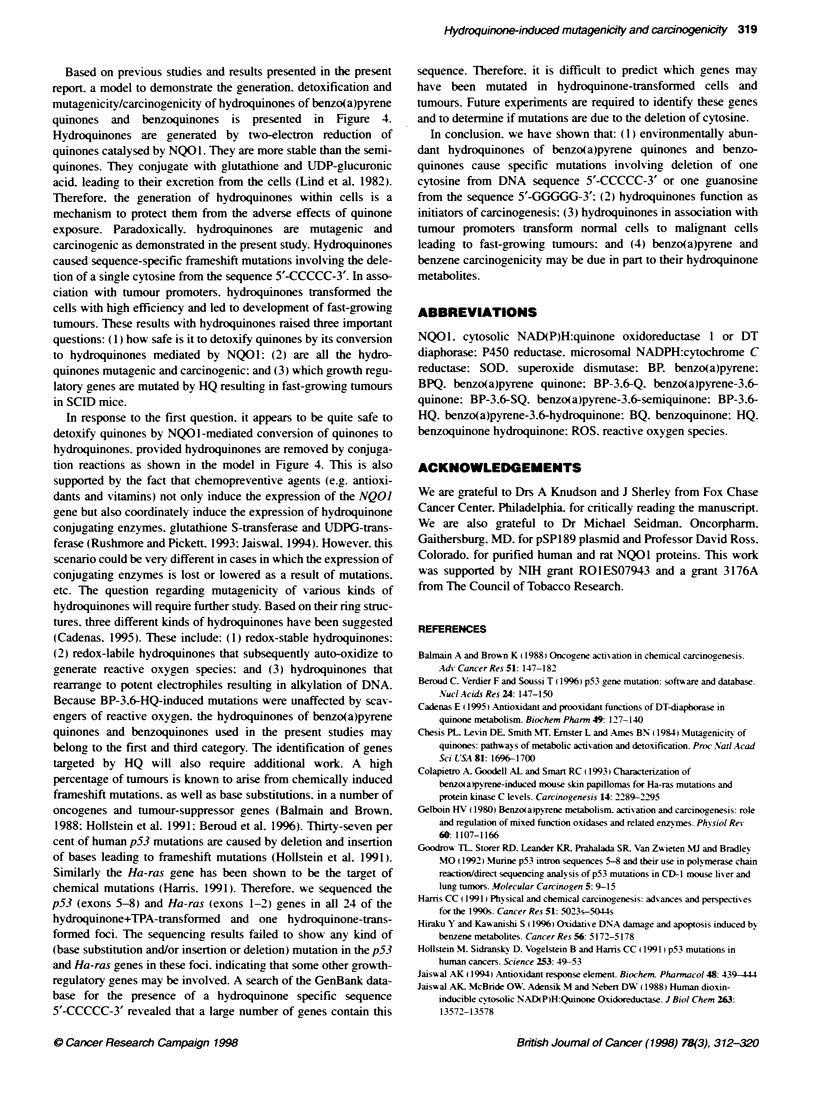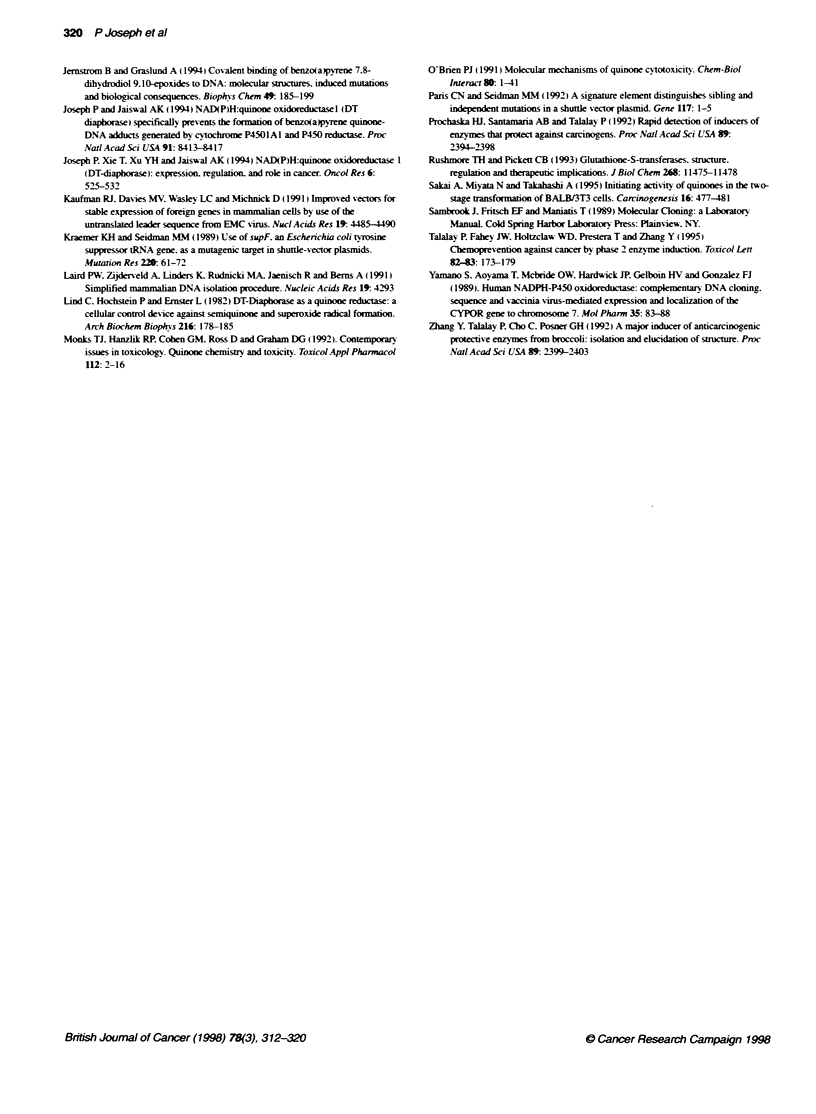# Hydroquinones cause specific mutations and lead to cellular transformation and in vivo tumorigenesis.

**DOI:** 10.1038/bjc.1998.492

**Published:** 1998-08

**Authors:** P. Joseph, A. J. Klein-Szanto, A. K. Jaiswal

**Affiliations:** Department of Pharmacology, Baylor College of Medicine, Houston, TX 77030, USA.

## Abstract

**Images:**


British Journal of Cancer (1998) 78(3), 312–320
© 1998 Cancer Research Campaign

### Hydroquinones cause specific mutations and lead to cellular transformation and in vivo tumorigenesis

**P Joseph[1], AJP Klein-Szanto[2] and AK Jaiswal[1]**

[1]Department of Pharmacology, Baylor College of Medicine, One Baylor Plaza, Houston TX 77030, USA; [2]Department of Pathology, Fox Chase Cancer Center, 7701 Burholme Avenue, Philadelphia PA 19111, USA

**Summary** Benzo(a)pyrene and benzene are human carcinogens. The metabolic activation of these compounds into ultimate mutagenic and carcinogenic metabolites is prerequisite for their carcinogenic effects. In this report, the mutagenicity and carcinogenicity of hydroquinones of benzo(a)pyrene and benzene was investigated to address two important questions: (1) do hydroquinones contribute to benzo(a)pyrene and benzene carcinogenicity; and (2) how safe is it to increase the levels of NAD(P)H:quinone oxidoreductase 1 (NQO1), a key enzyme in the generation of hydroquinone. The *supF* tRNA of the plasmid pSP189 was used as the mutational target in a cell-free and Chinese hamster ovary (CHO) cell system to study hydroquinone mutagenicity. RNA and protein-free pSP189 DNA was incubated in a cell-free system with benzo(a)pyrene-3,6-quinone and purified NQO1 or with benzoquinone hydroquinone to generate adducted pSP189 DNA. The adducted pSP189 DNA was transfected in human embryonic kidney cells Ad293. In the CHO cell system, monolayer cultures of CHO cells and CHO cells overexpressing NQO1 or P450 reductase were transfected with pSP189 vector DNA, treated with benzo(a)pyrene-3,6-quinone. The adducted and replicated pSP189 DNA was rescued from transfected Ad293 (cell-free system) and CHO cells (CHO cell system), digested with the restriction enzyme *Dpn*1 to remove unreplicated DNA followed by transformation in *Escherichia coli* MBM7070. The mutant colonies [white/pale blue on 5-bromo-4-chloro-3-indolyl β-D-galactoside/isopropyl β-D-thiogalactoside (X-gal/IPTG) plates] were selected, regrown and analysed by DNA sequencing. Mutagenesis experiments demonstrated that hydroquinones cause sequence-specific frameshift mutations involving deletion of a single cytosine from the DNA sequence 5′-172-CCCCC176-3′ or a single guanosine from the complementary strand sequence 5′-GGGGG-3′ in the *supF* tRNA gene. This mutation was specific to the hydroquinones, as it was not observed with quinones and other components of the redox cycling (semiquinones and reactive oxygen species). Exposure of BALBc/3T3 cells to hydroquinones resulted in cellular transformation leading to the loss of contact inhibition and regulation of cell growth. The transformation efficiency of BALBc/3T3 cells exposed to hydroquinones was significantly increased by the tumour promoter 12-*O*-tetradecanoylphorbol-13-acetate (TPA), indicating that hydroquinones are excellent initiators that require additional co-carcinogens or promoters to exert an effect. The hydroquinone + TPA as well as hydroquinone-transformed BALBc/3T3 cells, when injected s.c. in severe combined immunodeficient (SCID) mice, produced tumours at 100% frequency. These results establish that hydroquinones lead to mutagenicity and carcinogenicity.

**Keywords:** NAD(P)H:quinone oxidoreductase1 (DT diaphorase); hydroquinone; mutagenesis; cellular transformation; tumorigenesis; carcinogenesis

Exposure to environmental chemical carcinogens [e.g. benzo(a)pyrene and benzene] is known to cause numerous human cancers (Harris, 1991). Benzo(a)pyrene and benzene are procarcinogens that require metabolic activation to exert their mutagenic and carcinogenic effects (Gelboin, 1980). Benzo(a)pyrene undergoes oxidative metabolism to generate more than 25 metabolites (Gelboin, 1980). The most studied metabolite of benzo(a)pyrene is benzo(a)pyrene-7,8-dihydrodiol-9,10-epoxide (BPDE). BPDE is known to bind with DNA and cause G→T transversions, leading to carcinogenicity (Jernstrom and Graslund, 1994). These observations, although establishing an important role for BPDE in benzo(a)pyrene carcinogenicity, also raised interesting questions regarding the role of metabolites other than BPDE in benzo(a)pyrene mutagenicity and carcinogenicity. In addition, the metabolites of benzene responsible for benzene carcinogenicity remain to be identified. Oxidative metabolism of benzo(a)pyrene and benzene generates a common class of compounds, 'quinones' (Gelboin, 1980; O'Brien, 1991; Hiraku and Kawanishi, 1996). In addition to the benzo(a)pyrene quinones and benzoquinones, a variety of other quinones (e.g. naphthoquinones, tocopherol) are highly abundant in nature (Chesis et al, 1984). Therefore, human exposure to quinones is extensive. The quinones are highly reactive molecules that undergo further metabolism by one-step two-electron reduction [catalysed by NAD(P)H:quinone oxidoreductase1 (NQO1)] or two-step one-electron reduction [catalysed by NADPH:cytochrome P450 reductase (P450 reductase)] (O'Brien, 1991; Monks et al, 1992; Joseph et al, 1994; Talalay et al, 1995). The difference between the two-electron and one-electron reduction pathways for quinones is that the latter, and not the former, pathway generates semiquinones and reactive oxygen species of known toxicity and mutagenicity (O'Brien, 1991; Monks et al, 1992; Joseph et al, 1994; Talalay et al, 1995). For this reason the two-electron reduction pathway of conversion of quinones to hydroquinones by NQO1 is considered protective to the cells against the one-electron reduction pathway converting quinones to semiquinones and then to the hydroquinones. In fact, NQO1 has been shown to compete with P450 reductase for metabolic conversion of quinones to hydroquinone, resulting in protection to the

*Received 15 September 1997*
*Revised 7 January 1998*
*Accepted 20 January 1998*

*Correspondence to:* AK Jaiswal cells (Joseph and Jaiswal, 1994). The above observations have led to the discovery of natural and synthetic inducers of the *NQO1* gene, to increase the chemoprotective capacity of cells against exposure to quinones and related compounds (Prochaska et al, 1992; Zhang et al, 1992).

In this report, we have investigated the mutagenicity and carcinogenicity of hydroquinones to address two important questions: (1) do hydroquinones contribute to benzo(a)pyrene and benzene carcinogenicity; and (2) how safe is it to increase the levels of quinone detoxifying enzyme (NQO1), which catalyses conversion of quinones to hydroquinones. The various results demonstrated that hydroquinones specifically caused deletion of a single cytosine from the DNA sequence 5′-CCCCC-3′ of the *supF* tRNA gene. Hydroquinones also transformed BALBc/3T3 cells. The frequency of hydroquinone-induced transformation of BALBc/3T3 cells was significantly increased by tumour-promoting agent 12-*O*-tetradec anoylphorbol-13-acetate (TPA). The hydroquinone- and hydroquinone + TPA-transformed BALBc/3T3 cells, when injected s.c. in SCID mice, produced fast-growing tumours.

### MATERIALS AND METHODS

### Materials

The shuttle vector pSP189 carrying the mutational target *supF* tRNA gene was a generous gift from Dr Michael Seidman (Oncorpharm., Gaithersburg, MD, USA). The Chinese hamster ovary cells (CHO-DHFR$^-$), human embryonic kidney cells Ad293 and mouse fibroblast (BALBc/3T3) cells were obtained from ATCC, Rockville, MD, USA. The cDNA encoding P450 reductase was a kind gift from Dr Frank Gonzalez, NCI, Bethesda, MD, USA. Ingredients of the media used to grow the bacterial cells and to select the mutants were purchased from Difco Laboratories (Detroit, MI, USA). All other reagents used in the experiments were of the highest purity available commercially. The DNA sequencing kit version 2.0 was purchased from USB Corporation, Cleveland, OH, USA. BP-3,6-quinone was purchased from the Chemical Carcinogen Repository of the National Cancer Institute (Kansas City, MO, USA). Benzoquinone hydroquinone (HQ) was purchased from Sigma, St Louis, MO, USA. SCID mice were obtained from the Animal Facility at Fox Chase Cancer Center, Philadelphia, PA, USA.

Purified human and rat NQO1 were obtained as a gift from Dr David Ross, University of Colorado, Boulder, CO, USA. One unit of purified NQO1 activity is the amount of NQO1 protein that catalyses reduction of 1 μmol of 2,6-dichlorophenolindophenol in 1 min (Joseph and Jaiswal, 1994).

### Mutational analysis

#### *Cell-free system*

The *supF* tRNA of the plasmid pSP189 was used as the mutational target to study hydroquinone mutagenicity by procedures as described previously (Kraemer and Seidman, 1989; Paris and Seidman, 1992). Briefly, RNA and protein-free pSP189 DNA was prepared using the Qiagen plasmid preparation kit by the procedure as described in the manufacturer's instruction manual. The DNA was further cleaned with phenol–chloroform and ethanol precipitation by standard procedures (Sambrook et al, 1989). An aliquot of 10 μg of pSP189 DNA was incubated with 15 μM benzo(a)pyrene-3,6-quinone in the absence and presence of ten units of purified human or rat NQO1 activity under the conditions as described by us previously to generate BP-3,6-HQ and DNA adduct formation (Joseph and Jaiswal, 1994). In related experiments, the BP-3,6-Q was replaced with 15 μM hydroquinone (HQ) in the absence of NQO1 enzyme. The adducted pSP189 DNA was isolated and purified by the phenol–chloroform extraction and ethanol precipitation procedure and used to transfect human embryonic kidney cells Ad293 by the calcium phosphate precipitation procedure (Sambrook et al, 1989). pSP189 DNA rescued from the transfected Ad293 cells was digested with the restriction enzyme DpnI to remove unreplicated DNA and was used to transform competent *E scherichia coli* strain MBM7070 by electroporation using a Gene Pulser apparatus (Bio Rad, Hercules, CA) set at 2.5 kV, 25 μF and 200 Ω according to the manufacturer's instructions. Mutants growing as white or pale blue colonies on LB plates containing 50 μg ml$^{-1}$ ampicillin, 20 μg ml$^{-1}$ 5-bromo-4-chloro-3-indolyl β-D-galactoside (X-gal) and 100 μg ml$^{-1}$ isopropyl β-D-thiogalactoside (IPTG) were selected and regrown on fresh plates. The plasmid DNA from the mutants was isolated and purified by the alkaline lysis procedure (Sambrook et al, 1989). The *SupF* tRNA region of pSP189 isolated from mutant colonies was sequenced using the sequenase version 2.0 DNA sequencing kit. It may be noteworthy that every mutation was selected from a single transformation to avoid sibling formation.

#### *Chinese hamster ovary (CHO) cell system*

***Development of CHO cells permanently expressing high levels of microsomal P450 reductase or cytosolic NQO1 activity.*** Human cDNAs encoding cytosolic NQO1 and microsomal P450 reductase have been cloned and sequenced (Jaiswal et al, 1988; Yamano et al, 1989). Human cDNAs for NQO1 and P450 reductase were separately subcloned in pED6 vector to generate pED6-NQO1 and pED6-P450 reductase plasmids (Kaufman et al, 1991). The CHO cells were transfected with pED6-P450 reductase or pED6-NQO1 recombinant plasmid and selected in the presence of increasing concentrations of methotrexate by procedures as described previously (Kaufman et al, 1991). The selected clones were analysed for NQO1 and P450 reductase activities by procedures as described previously (Joseph and Jaiswal, 1994). The clone designated as CHO (NQO1) selected for overexpression of cDNA-derived NQO1 expressed 1367-fold higher levels of NQO1 activity than the untransfected/vector transfected control CHO cells. Similarly, the selected clone CHO (P450 reductase) expressed 34-fold higher levels of cDNA-derived microsomal P450 reductase as compared with control CHO cells. The control CHO (wild type), CHO (NQO1) and CHO (P450 reductase) were used in hydroquinone mutagenesis studies.

Monolayers of CHO (wild type), CHO (NQO1) and CHO (P450 reductase) were transfected with pSP189 vector DNA by the calcium phosphate co-precipitation procedure (Kraemer and Seidman, 1989). The transfected cells were grown in the medium containing 30 μM BP-3,6-Q for 4 h. Forty-eight hours following the transfection, the shuttle vector DNA was rescued and the *supF* tRNA gene analysed for mutation as described under the in vitro mutation studies.

### Hydroquinone carcinogenicity

#### *Transformation of BALBc/3T3 cells*

The transformation of BALBc/3T3 cells by hydroquinone was studied by a previously described procedure (Sakai et al, 1995).

© Cancer Research Campaign 1998

**Table 1** Frequency of benzo(a)pyrene-3.6-hydroquinone (BP-3.6-HQ)- and benzoquinone hydroquinone (HQs)-induced deletion of a single cytosine from sequence 5′-172-CCCCC-176-3′ of the pSP189.

| Sample | Total mutation frequency per million transformants[a] | Mutation frequency of deletion of a single cytosine from sequence 5′-172-CCCCC-176-3′/ million transformants[b] |
|---|---|---|
| *Cell-free system*[c] | | |
| pSP189 + DMSO | 1.11 ± 0.02 | 0.0 |
| pSP189 + BP-3.6-Q | 2.75 ± 0.05 | 0.0 |
| pSP189 + BP-3.6-Q + P450 reductase | 5.95 ± 0.39 | 0.0 |
| pSP189 + BP-3.6-Q + purified human NQO1 | 2.38 ± 0.11 | 0.30 ± 0.02 |
| pSP189 + BP-3.6-Q + purified rat NQO1 | 2.11 ± 0.17 | 0.37 ± 0.03 |
| pSP189 + BP-3.6-Q + purified human NQO1 + SOD (30U) + catalase (40U) | 2.41 ± 0.36 | 0.30 ± 0.03 |
| pSP189 + HQ | 2.28 ± 0.41 | 0.18 ± 0.02 |
| *CHO cell system*[c] | | |
| CHO (wild type) + pSP189 + DMSO | 1.31 ± 0.06 | 0.0 |
| CHO (wild type) + pSP189 + BP-3.6-Q | 2.41 ± 0.29 | 0.0 |
| CHO (NQO1) + pSP189 + DMSO | 1.21 ± 0.23 | 0.0 |
| CHO (NQO1) + pSP189 + BP-3.6-Q | 2.40 ± 0.31 | 0.24 ± 0.03 |
| CHO (P450 reductase)[c] + pSP189 + BP-3.6-Q | 5.71 ± 0.69 | 0.0 |

[a]Total mutation frequencies were determined by counting the number of mutant colonies observed per million transformants. The results are presented as mean ± s.e. of four independent experiments. [b]Total of 40 mutants were sequenced in each set. The results are presented as mean ± s.e. of four independent experiments. Note that mutational spectra other than deletion of a single cytosine in all the cases were similar to the spontaneous mutations and are shown in Table 2. [c]Cell-free system. The plasmid pSP189 containing the mutational target *supF* tRNA gene was incubated with DMSO (spontaneous mutations) or BP-3.6-Q (15 μM) in absence and presence of purified human and rat NQO1 (5 μg) and superoxide dismutase (SOD) and catalase as indicated. In related experiments, the BP-3.6-Q was replaced with 15 μM HQ. Hydroquinone-induced mutations in the *supF* tRNA region were determined by procedures as described in Materials and methods. The purified human and rat NQO1 were obtained from Dr David Ross, School of Pharmacy, Denver, CO, USA. Both human and rat NQO1 enzymes are known to catalyse high-affinity reduction of BP-3, 6-Q to BP-3,6-HQ. [d]CHO cell system. The Chinese hamster ovary (CHO) cells (wild type) expressing endogenous levels of cytosolic NQO1 and microsomal P450 reductase and the CHO cells permanently expressing either 1367-fold higher level of cDNA derived cytosolic NQO1 (NQO1) or 34-fold higher levels of cDNA derived microsomal P450 reductase (P450 reductase) were transfected with pSP189 plasmid in separate experiments. The transfected cells were treated with DMSO (control) or with BP-3.6-Q. NQO1 catalyses two-electron reduction of BP-3.6-Q (quinone) to BP-3.6-HQ (hydroquinone). P450 reductase catalyses one-electron reductive activation of BP-3.6-Q (quinone) to BP-3.6-SQ (semiquinone) and ROS (reactive oxygen species). The metabolites thus generated bind to the plasmid pSP189, resulting in the formation of DNA adducts leading to mutagenicity. Mutational spectra in each case was determined as described in the text.

Briefly, BALBc/3T3 cells were grown as a monolayer in Dulbecco's modified Eagle's medium (DMEM) containing 10% calf serum. One day before the initiation of transformation, 16 000 cells were plated per 100-mm Petri dish (40 plates per group). The cells were allowed to grow in the medium containing 15 μM hydroquinone for 3 days. The medium was changed and the cells were grown in fresh medium for 3 days. Subsequently, the cells were grown in the medium containing 300 ng $ml^{-1}$ TPA for 2 weeks, during which period the medium was changed twice weekly. The cells were allowed to grow in the control medium for an additional 2 weeks and the individually growing transformed foci (>3 mm size) were counted. It may be noteworthy that not more than one focus was visible per plate. Therefore, the various foci selected by us represent independent clones of transformed BALBc/3T3 cells and the question of siblings does not arise.

### *Growth of normal and HQ+TPA transformed BALBc/3T3 cells*

A similar number of normal and HQ+TPA-transformed BALBc/3T3 cells were plated in separate Petri dishes. The cells were grown under similar (5% $CO_2$; 37°C) conditions. Four days after growth, the cells were photographed and growth of normal and transformed cells compared.

### *Subcutaneous injection of HQ- and HQ+TPA-transformed BALBc/3T3 cells and development of tumours in SCID mice*

All the foci representing various clones of HQ- and HQ+TPA-transformed BALBc/3T3 cells were tested for their capacity to form tumours in SCID mice. In each case, $10 \times 10^6$ cells in a volume of 200 μl growth medium were subcutaneously injected into the SCID mice. Appropriate controls received either the growth medium or the control BALBc/3T3 cells. The mice were observed for the development of subcutaneous tumour at the site of the injection for a period of 4 weeks.

### *Analysis of* p53 *and* Ha-ras *genes in the transformed BALBc/3T3 foci*

The HQ- and HQ+TPA-transformed foci of the BALBc/3T3 cells were individually transferred into 24-well plates and further expanded. Genomic DNA from the various foci was isolated by the procedure as described (Laird et al. 1991). This DNA was used as template to amplify the p53 (exons 5–8) and the *Ha-ras* (exons 1 and 2) genes using polymerase chain reaction (PCR). Exons 5–8 of the p53 tumour suppressor-gene were sequenced individually using the exon-based primers under the conditions as described (Goodrow et al. 1992). Exons 1 and 2 of the *Ha-ras* oncogene were amplified as a single fragment by a modification of the previously

© Cancer Research Campaign 1998

**Table 2** Mutations of the *SupF* tRNA gene (pSP189–Ad293 cells)

| | pSP189 + DMSO | pSP189 + BPQ | pSP189 + BPQ + Reductase | pSP189 + BPQ + HDTD | pSP189 + BPQ + HDTD + SOD + Catalase | pSP189 + BPQ + RDTD | pSP189 + HQ |
|---|---|---|---|---|---|---|---|
| No. of colonies analysed | 40 | 40 | 40 | 40 | 40 | 40 | 40 |
| No. of mutants | 40 | 40 | 40 | 38 | 40 | 40 | 38 |
| *Mutations* | | | | | | | |
| Base substitutions | | | | | | | |
| A→C | 2 | 3 | 4 | 3 | 3 | 1 | 3 |
| A→T | 4 | 2 | 1 | 4 | 2 | 3 | 2 |
| A→G | 2 | 3 | 2 | 0 | 1 | 2 | 2 |
| C→A | 1 | 4 | 2 | 2 | 1 | 1 | 2 |
| C→T | 3 | 2 | 1 | 0 | 1 | 0 | 0 |
| C→G | 2 | 3 | 2 | 2 | 2 | 2 | 2 |
| T→A | 3 | 1 | 1 | 2 | 3 | 2 | 3 |
| T→C | 3 | 2 | 1 | 2 | 1 | 3 | 1 |
| T→G | 2 | 2 | 1 | 0 | 2 | 2 | 1 |
| G→A | 3 | 1 | 4 | 1 | 4 | 3 | 2 |
| G→C | 2 | 3 | 3 | 2 | 1 | 2 | 2 |
| G→T | 3 | 1 | 3 | 2 | 2 | 1 | 4 |
| Deletions | | | | | | | |
| A | 1 | 2 | 1 | 2 | 2 | 1 | 2 |
| C (Random) | 0 | 0 | 3 | 2 | 2 | 3 | 2 |
| **C (172-CCCCC-176)** | **0** | **0** | **0** | **5** | **7** | **5** | **3** |
| T | 0 | 1 | 0 | 0 | 0 | 2 | 0 |
| G | 1 | 2 | 5 | 2 | 1 | 1 | 1 |
| 68–119 | 3 | 3 | 1 | 1 | 2 | 2 | 2 |
| Insertions | | | | | | | |
| A | 1 | 0 | 0 | 1 | 0 | 1 | 0 |
| T | 0 | 0 | 1 | 1 | 0 | 0 | 0 |
| Unclassified | 4 | 5 | 4 | 4 | 3 | 3 | 4 |

Mutation frequencies of benzo(a)pyrene-3,6-quinone (BP-3,6-Q); benzo(a)pyrene-3,6-semiquinone + reactive oxygen species (BP-3,6-SQ + ROS) and benzo(a)pyrene-3,6-hydroquinone (BP-3,6-HQ) in a cell-free system. The plasmid pSP189 containing the mutational target *SupF* tRNA was incubated with DMSO (spontaneous mutations) or with COS1 cell extract expressing 68-fold higher levels of cDNA-derived cytochrome P450 reductase (BP-3,6-semiquinone + ROS induced mutations) or with purified human or rat NQO1 (hydroquinone-induced mutations). The experiment with purified NQO1 (hydroquinone mutagenicity) was also performed in the presence of SOD and catalase. Mutations in each case were detected by the procedures as described in Materials and methods. A total of 40 mutants was sequenced in each set. Frequency of mutations are shown per million transformants. HDTD, human DT diaphorase (NQO1); RDTD, rat DT diaphorase (NQO1).

described procedure using the primers and PCR conditions as given below (Colapietro et al, 1993):

Primer 1, 5′-ATCACAGAATACAAGCTTGTGG-3′
Primer 2, 5′-CTGTACTGATGGATGTCCTC-3′

The PCR conditions involved 30 cycles of denaturation at 94°C for 1 min, annealing at 58°C for 2 min and extension at 72°C for 2 min followed by an extension of 7 min at 72°C.

The PCR products were analysed on 1% agarose gel and subcloned immediately into the PCR 2.1 vector of the TA cloning kit (Invitrogen, San Diego, CA, USA) and used to transform competent *E. coli* cells according to the protocols of the manufacturer. DNA was isolated from the transformants and sequenced using the sequenase version 2.0 DNA sequencing kit. The primers used to sequence the exon 1 and exon 2 of the *Ha-ras* gene were the same as reported (Colapietro et al, 1993).

### RESULTS

The results on BP-3,6-HQ- and HQ-induced mutations from the studies using a cell-free system are reported in Tables 1 and 2. The spontaneous mutation frequency of pSP189 plasmid DNA treated with DMSO was 1.11±0.02 per million transformants in the cell-free system and 1.31±0.06 per million transformants in the CHO cell system (Table 1). The total mutation frequencies in both the systems increased twofold in the presence of BP-3,6-Q. P450 reductase but not NQO1 further increased the total mutation frequency by 2.5-fold, as compared with BP-3,6-Q (Table 1). Inclusion of superoxide dismutase and catalase (scavengers of ROS) with NQO1 had no effect on total mutation frequency observed with BP-3,6-Q + NQO1 (Table 1). Analysis of the mutation spectra revealed that all the mutations except deletion of one cytosine from the sequence 5′-172-CCCCC-3′ were more or less similar to the spontaneous (DMSO) mutations. It may be noteworthy that hydroquinone-induced deletion of a single cytosine may have been the result of deletion of a single guanosine in the complementary strand. The deletion of cytosine from a stretch of five cytosines was not observed in spontaneous mutations and in the mutations caused by quinone (BP-3,6-Q) and P450 reductase-activated quinone BP-3,6-Q (BP-3, 6-semiquinone and reactive oxygen species) (Figure 1, Tables 1 and 2). However, the deletion of one cytosine from 5′-CCCCC-3′ was observed with BP-3,6-HQ.

**Figure 1** (**A**) Nucleotide sequence of *supF* tRNA gene in pSP189. Hot spot for hydroquinone (HQ)-induced frameshift mutation involving deletion of a single cytosine is indicated in bold letters. (**B**) Hydroquinone (HQ)-induced deletion of a single cytosine from sequence 5′-CCCCC-3′. Left. DNA sequence in 5′→3′ orientation. Note five Cs vs four Cs. Right. DNA sequence in 3′→5′ orientation. Note five Gs versus four Gs

The mutation frequency of deletion of cytosine induced by BP-3,6-HQ was 0.30 (12.6% of total mutation frequency) with human NQO1 enzyme and 0.37 (17.5% of total mutation frequency) with rat NQO1 enzyme per million transformants (Table 1). These mutation frequencies of deletion of one cytosine from five cytosines were highly significant because the background (spontaneous mutation) frequency was zero. The deletion of one cytosine from the sequence 5′-CCCCC-3′ was also specific to the hydroquinones (BP-3,6-HQ) because similar mutations were not observed with quinones (BP-3,6-Q) and redox cycling products of quinones (semiquinones + reactive oxygen species). Interestingly, the mutation frequency of deletion of cytosine remained more or less unaffected in the presence of high amounts of purified superoxide dismutase (SOD) and catalase, the scavengers of reactive oxygen species. A second hydroquinone (benzoquinone HQ) caused similar cytosine deletion mutations as BP-HQ at mutation frequency of 0.18 per million transformants (Tables 1 and 2). The nucleotide sequence of *supF* tRNA region of plasmid pSP189 and the HQ-induced deletion of one cytosine from a stretch of five cytosines are shown in Figure 1.

Similar results as described above with the cell-free system were also observed in in vivo mutagenesis experiments as shown in Tables 1 and 3. The CHO (wild type) cells expressing low (endogenous) levels of NQO1 and P450 reductase, the CHO (NQO1) cells permanently expressing 1367-fold higher levels of cDNA-derived human NQO1 and the CHO (P450 reductase) cells permanently expressing 34-fold higher levels of cDNA-derived human P450 reductase were transfected with pSP189 plasmid followed by treatment with BP-3,6-Q. Mutational spectra in each case were determined by standard procedures (Kraemer and Seidman, 1989; Pairs and Seidman, 1992). The treatment of CHO cells expressing endogenous levels of NQO1 and P450 reductase

**Figure 2** Comparison of growth of normal and HQ+TPA-transformed BALBc/3T3 cells. Three hundred normal and a similar number of HQ+TPA-transformed cells were plated and grown under similar conditions. After four days, the cells in Petri dishes were visualized under microscope and photographed. (**A**) Normal (DMSO-treated) cells. (**B**) HQ+TPA-transformed cells and the CHO cells expressing high levels of P450 reductase failed to show the deletion of cytosine from sequence 5′-172-CCCCC176-3′. However, the CHO cells expressing higher levels of NQO1 showed deletion of a single cytosine at a frequency of 10% of total mutations or mutation frequency of 0.24 per million transformants.

Incubation of $0.64 \times 10^6$ mouse BALBc/3T3 cells with benzoquinone hydroquinone (HQ) resulted in the transformation of BALBc/3T3 cells and selection of a single cloned focus (Table 4). However, incubation of a similar number of BALBc/3T3 cells with hydroquinone, followed by tumour promoter TPA, significantly increased the number of transformed foci to 24 (Table 4). In the same experiment, TPA alone failed to generate any foci. The 24 foci selected with hydroquinone+TPA treatment grew 10–20 times faster than normal (untransformed) BALBc/3T3 cells and lost contact inhibition, as demonstrated in Figure 2. These results also indicated that hydroquinones may function as initiators of carcinogenesis and require a promoter (e.g. TPA) for cellular transformation and proliferation. It may be noteworthy that in a similar experiment, 3-methylcholanthrene (3-MC) also transformed BALBc/3T3 cells and produced 22 foci (Table 4 legend). Inclusion of TPA in the experiment increased the number of 3-MC induced foci to 47.

 © Cancer Research Campaign 1998

**Table 3** Mutations of the *SupF* tRNA gene (pSP 189 – CHO cells)

| | CHO (W) + pSP189 + DMSO | CHO (W) + pSP189 + BPQ | CHO (NQO1) + pSP189 + DMSO | CHO (NQO1) pSP189 + BPQ | CHO (RED) + pSP189 + BPQ |
|---|---|---|---|---|---|
| No. of coloniesanalysed | 40 | 40 | 40 | 40 | 40 |
| No. of mutants | 40 | 40 | 38 | 40 | 40 |
| *Mutations* | | | | | |
| Base substitutions | | | | | |
| A→C | 4 | 2 | 3 | 3 | 2 |
| A→T | 3 | 4 | 2 | 3 | 1 |
| A→G | 3 | 1 | 2 | 2 | 3 |
| C→A | 1 | 3 | 3 | 2 | 2 |
| C→T | 2 | 1 | 2 | 1 | 2 |
| C→G | 2 | 2 | 3 | 2 | 2 |
| T→A | 3 | 3 | 2 | 3 | 2 |
| T→C | 3 | 3 | 2 | 1 | 1 |
| T→G | 2 | 4 | 2 | 3 | 2 |
| G→A | 4 | 2 | 1 | 3 | 3 |
| G→C | 2 | 1 | 2 | 0 | 3 |
| G→T | 3 | 2 | 3 | 2 | 1 |
| Deletions | | | | | |
| A | 1 | 3 | 2 | 3 | 4 |
| C (Random) | 0 | 1 | 0 | 2 | 0 |
| **C (172-CCCCC-176)** | **0** | **0** | **0** | **4** | **0** |
| T | 0 | 0 | 2 | 0 | 1 |
| G | 2 | 1 | 1 | 0 | 6 |
| 68–119 | 2 | 2 | 3 | 4 | 2 |
| Insertions | | | | | |
| A | 0 | 1 | 0 | 0 | 0 |
| C | 0 | 0 | 0 | 0 | 1 |
| Unclassified | 3 | 4 | 3 | 2 | 2 |

Mutation frequencies of benzo(a)pyrene-3.6-quinone (BP-3.6-Q). benzo(a)pyrene-3.6-hydroquinone (BP-3.6-HQ) and benzo(a)pyrene-3.6-semiquinone + reactive oxygen species (BP-3.6-SQ + ROS) as determined by using CHO cell system. The Chinese hamster ovary (CHO) cells (wild type). CHO cells expressing 1367-fold higher levels of cDNA-derived human NQO1 and CHO cells expressing 68-fold higher levels of cytochrome P450 reductase were transfected with plasmid pSP189. The transfected cells were treated with either DMSO (control) or benzo(a)pyrene-3.6-quinone (BP-3.6-Q). The mutational spectra generated due to DMSO (spontaneous) and metabolites of BP-3.6-Q were determined by procedures as described in Materials and methods. Mutation frequencies are shown per million transformants.

Interestingly. all of the 24 BALBc/3T3 foci selected with hydroquinone+TPA produced subcutaneous tumours in SCID mice (Figure 3). Twenty per cent of the hydroquinone+TPA-transformed foci grew very fast and tumours became visible after 1 week. In the remaining 80% of cases. the tumours were visible between 2 and 4 weeks. A single hydroquinone-transformed colony of BALBc/3T3 cells also produced tumours in SCID mice that were visible after 4 weeks of injection. Under similar conditions. control BALBc/3T3 cells did not produce tumours in SCID mice in 24 weeks of our observation.

### DISCUSSION

In this report. we demonstrate that hydroquinones of benzo(a)pyrene quinones and benzoquinones (BP-HQ and HQ) are mutagenic compounds. Mutagenesis experiments clearly indicate that BP-HQ and HQ both cause sequence-specific deletion of a single cytosine from a group of five cytosines or a single guanosine from a group of five guanosines in the complementary strand. resulting in frameshift mutations. This type of frameshift mutation was not detected in spontaneous mutations. mutations caused by quinones or mutations generated by semiquinones and reactive oxygen species. two important products of the redox cycling of

**Figure 3** Hydroquinone-induced subcutaneous tumours. Groups of ten mice were subcutaneously injected with either control (normal) BALBc/3T3 cells or HQ-and HQ+TPA-transformed BALBc/3T3 cells. Each mouse received ten million cells in growth medium. The mice were observed for the development of subcutaneous tumours at the site of injection. Mice injected with control (normal) cells did not produce tumours as shown on the left. Mice injected with hydroquinone +TPA produced fast-growing tumours as shown on the right. The position of the tumour in the mouse on the right side is indicated by an arrow. Twenty out of 24 hydroquinone+TPA-transformed BALBc/3T3 foci produced fast-growing tumours in SCID mice. The remaining four hydroquinone+TPA- and a single hydroquinone-transformed BALBc/3T3 foci produced slow-growing tumors. which became visible after 8 weeks (data not shown)

Table 4 Hydroquinone-induced transformation of BALBc/3T3 cells[a]

| Chemical | Number of foci |
|---|---|
| Ethanol | 0 |
| HQ | 1 |
| HQ + TPA | 24 |
| TPA | 0 |

[a] 0.64×10[6] BALBc/3T3 cells were plated at a density of 16 000 cells in 100 mm Petri dishes. The cells were exposed for 72 h either to 15 μM benzoquinone hydroquinone (HQ) or to ethanol, used as the solvent to dissolve hydroquinone. The cells were washed and allowed to grow in the control medium for 3 days. Subsequently, the cells were exposed either to 12-O-tetradecanoylphorbol-13-acetate (TPA; 300 ng ml⁻¹ of the medium) or to acetone (the solvent used to dissolve TPA) for 2 weeks. The cells were washed to remove TPA and were allowed to grow for an additional 2 weeks in the control medium. The foci developed were scored, isolated and expanded individually. Under similar experimental conditions as described above, 3-methycholanthrene (3-MC) produced 22 foci with 3-MC alone and 47 foci with 3-MC + TPA treatment.

quinones. We further demonstrated that BP-HQ-induced deletion of a single cytosine from sequence 5′-CCCCC-3′ was not mediated by reactive oxygen species generated by oxidation of BP-HQ. This is because the mutation frequency of deletion of a single cytosine was not affected by SOD and catalase, well-known scavengers of the reactive oxygen species. It is expected that BP-HQ may directly alkylate the DNA at specific sites containing a stretch of five cytosines or five guanosines leading to deletion of a single cytosine or guanosine by an unknown mechanism.

Several observations indicate that DNA sequences containing 5′-CCCCC-3′ or 5′-GGGGG-3′ serve as hot spots for BP-HQ and HQ binding and mutagenicity. Firstly, the only mutation detected with BP-HQ and HQ above the level of spontaneous mutations was deletion of a single cytosine from sequence 5′-CCCCC-3′. The stretch of four cytosines and guanosines within the *supF* tRNA region was not targeted by BP-HQ and HQ. Secondly, two different hydroquinones (BP-HQ and HQ) showed similar mutations involving deletion of a single cytosine with significant frequencies (Table 1). Lastly, hydroquinone-induced deletion of 'C' from sequence 5′-CCCCC-3′ was not detected in spontaneous mutations and in mutations induced by unmetabolized quinones and P450 reductase-activated products of quinones (semiquinones and reactive oxygen species).

The HQ treatment of BALBc/3T3 cells resulted in cellular transformation, which was significantly increased by tumour-promoting agents (TPA). The hydroquinone + TPA-transformed BALBc/3T3 cells produced fast-growing tumours in SCID mice. These results suggested that hydroquinones possess initiating capacity and require tumour promoters for complete transformation of cells leading to the development of fast-growing tumours. The various results also suggest that hydroquinone possesses much weaker initiating activity than 3-MC.

Figure 4 Model for benzo(a)pyrene hydroquinone and benzoquinone hydroquinone mutagenicity and carcinogenicity. Benzo(a)pyrene quinones and benzoquinones either directly or after reductive metabolism by P450 reductase into products (semiquinones and reactive oxygen species) bind with the DNA and cause mutations. On the other hand, NQO1 competes with P450 reductase and catalyses the formation of hydroquinones. It may be noteworthy that semiquinones generated during P450 reductase-stimulated redox cycling may also be converted to hydroquinone by a second electron reduction. This is especially the case in the absence of oxygen. Hydroquinones conjugate with UDP-glucuronic acid, glutathione and sulphate, leading to their excretion from the cells. The various conjugation reactions are catalysed by UDPG-transferase (UDPGT), glutathione S-transferase (GST) and sulphotransferase (ST). Therefore, the NQO1 pathway protects the cells from adverse effects of semiquinones and reactive oxygen species by preventing their formation. However, hydroquinones produced during the metabolic reduction of quinones by NQO1, if not conjugated with glutathione or UDP-glucuronic acid and excreted from the cells, cause frameshift mutations involving deletion of a single cytosine from sequence 5′-CCCCC-3′. This type of mutation was not observed with quinones, semiquinones and reactive oxygen species. Therefore, the deletion mutation of a single cytosine from sequence 5′-CCCCC-3′ is specifically associated with hydroquinones. In addition, the hydroquinones in association with tumour promoters transformed normal cells to malignant cells leading to tumour formation

 © Cancer Research Campaign 1998

Based on previous studies and results presented in the present report, a model to demonstrate the generation, detoxification and mutagenicity/carcinogenicity of hydroquinones of benzo(a)pyrene quinones and benzoquinones is presented in Figure 4. Hydroquinones are generated by two-electron reduction of quinones catalysed by NQO1. They are more stable than the semiquinones. They conjugate with glutathione and UDP-glucuronic acid, leading to their excretion from the cells (Lind et al, 1982). Therefore, the generation of hydroquinones within cells is a mechanism to protect them from the adverse effects of quinone exposure. Paradoxically, hydroquinones are mutagenic and carcinogenic as demonstrated in the present study. Hydroquinones caused sequence-specific frameshift mutations involving the deletion of a single cytosine from the sequence 5′-CCCCC-3′. In association with tumour promoters, hydroquinones transformed the cells with high efficiency and led to development of fast-growing tumours. These results with hydroquinones raised three important questions: (1) how safe is it to detoxify quinones by its conversion to hydroquinones mediated by NQO1; (2) are all the hydroquinones mutagenic and carcinogenic; and (3) which growth regulatory genes are mutated by HQ resulting in fast-growing tumours in SCID mice.

In response to the first question, it appears to be quite safe to detoxify quinones by NQO1-mediated conversion of quinones to hydroquinones, provided hydroquinones are removed by conjugation reactions as shown in the model in Figure 4. This is also supported by the fact that chemopreventive agents (e.g. antioxidants and vitamins) not only induce the expression of the *NQO1* gene but also coordinately induce the expression of hydroquinone conjugating enzymes, glutathione S-transferase and UDPG-transferase (Rushmore and Pickett, 1993; Jaiswal, 1994). However, this scenario could be very different in cases in which the expression of conjugating enzymes is lost or lowered as a result of mutations, etc. The question regarding mutagenicity of various kinds of hydroquinones will require further study. Based on their ring structures, three different kinds of hydroquinones have been suggested (Cadenas, 1995). These include: (1) redox-stable hydroquinones; (2) redox-labile hydroquinones that subsequently auto-oxidize to generate reactive oxygen species; and (3) hydroquinones that rearrange to potent electrophiles resulting in alkylation of DNA. Because BP-3,6-HQ-induced mutations were unaffected by scavengers of reactive oxygen, the hydroquinones of benzo(a)pyrene quinones and benzoquinones used in the present studies may belong to the first and third category. The identification of genes targeted by HQ will also require additional work. A high percentage of tumours is known to arise from chemically induced frameshift mutations, as well as base substitutions, in a number of oncogenes and tumour-suppressor genes (Balmain and Brown, 1988; Hollstein et al, 1991; Beroud et al, 1996). Thirty-seven per cent of human *p53* mutations are caused by deletion and insertion of bases leading to frameshift mutations (Hollstein et al, 1991). Similarly the *Ha-ras* gene has been shown to be the target of chemical mutations (Harris, 1991). Therefore, we sequenced the *p53* (exons 5–8) and *Ha-ras* (exons 1–2) genes in all 24 of the hydroquinone+TPA-transformed and one hydroquinone-transformed foci. The sequencing results failed to show any kind of (base substitution and/or insertion or deletion) mutation in the *p53* and *Ha-ras* genes in these foci, indicating that some other growth-regulatory genes may be involved. A search of the GenBank database for the presence of a hydroquinone specific sequence 5′-CCCCC-3′ revealed that a large number of genes contain this sequence. Therefore, it is difficult to predict which genes may have been mutated in hydroquinone-transformed cells and tumours. Future experiments are required to identify these genes and to determine if mutations are due to the deletion of cytosine.

In conclusion, we have shown that: (1) environmentally abundant hydroquinones of benzo(a)pyrene quinones and benzoquinones cause specific mutations involving deletion of one cytosine from DNA sequence 5′-CCCCC-3′ or one guanosine from the sequence 5′-GGGGG-3′; (2) hydroquinones function as initiators of carcinogenesis; (3) hydroquinones in association with tumour promoters transform normal cells to malignant cells leading to fast-growing tumours; and (4) benzo(a)pyrene and benzene carcinogenicity may be due in part to their hydroquinone metabolites.

### ABBREVIATIONS

NQO1, cytosolic NAD(P)H:quinone oxidoreductase 1 or DT diaphorase; P450 reductase, microsomal NADPH:cytochrome *C* reductase; SOD, superoxide dismutase; BP, benzo(a)pyrene; BPQ, benzo(a)pyrene quinone; BP-3,6-Q, benzo(a)pyrene-3,6-quinone; BP-3,6-SQ, benzo(a)pyrene-3,6-semiquinone; BP-3,6-HQ, benzo(a)pyrene-3,6-hydroquinone; BQ, benzoquinone; HQ, benzoquinone hydroquinone; ROS, reactive oxygen species.

### ACKNOWLEDGEMENTS

We are grateful to Drs A Knudson and J Sherley from Fox Chase Cancer Center, Philadelphia, for critically reading the manuscript. We are also grateful to Dr Michael Seidman, Oncorpharm, Gaithersburg, MD, for pSP189 plasmid and Professor David Ross, Colorado, for purified human and rat NQO1 proteins. This work was supported by NIH grant RO1ES07943 and a grant 3176A from The Council of Tobacco Research.

### REFERENCES

Balmain A and Brown K (1988) Oncogene activation in chemical carcinogenesis. *Adv Cancer Res* **51**: 147–182

Beroud C, Verdier F and Soussi T (1996) p53 gene mutation: software and database. *Nucl Acids Res* **24**: 147–150

Cadenas E (1995) Antioxidant and prooxidant functions of DT-diaphorase in quinone metabolism. *Biochem Pharm* **49**: 127–140

Chesis PL, Levin DE, Smith MT, Ernster L and Ames BN (1984) Mutagenicity of quinones: pathways of metabolic activation and detoxification. *Proc Natl Acad Sci USA* **81**: 1696–1700

Colapietro A, Goodell AL and Smart RC (1993) Characterization of benzo(a)pyrene-induced mouse skin papillomas for Ha-ras mutations and protein kinase C levels. *Carcinogenesis* **14**: 2289–2295

Gelboin HV (1980) Benzo(a)pyrene metabolism, activation and carcinogenesis: role and regulation of mixed function oxidases and related enzymes. *Physiol Rev* **60**: 1107–1166

Goodrow TL, Storer RD, Leander KR, Prahalada SR, Van Zwieten MJ and Bradley MO (1992) Murine p53 intron sequences 5–8 and their use in polymerase chain reaction/direct sequencing analysis of p53 mutations in CD-1 mouse liver and lung tumors. *Molecular Carcinogen* **5**: 9–15

Harris CC (1991) Physical and chemical carcinogenesis: advances and perspectives for the 1990s. *Cancer Res* **51**: 5023s–5044s

Hiraku Y and Kawanishi S (1996) Oxidative DNA damage and apoptosis induced by benzene metabolites. *Cancer Res* **56**: 5172–5178

Hollstein M, Sidransky D, Vogelstein B and Harris CC (1991) p53 mutations in human cancers. *Science* **253**: 49–53

Jaiswal AK (1994) Antioxidant response element. *Biochem. Pharmacol* **48**: 439–444

Jaiswal AK, McBride OW, Adensik M and Nebert DW (1988) Human dioxin-inducible cytosolic NAD(P)H:Quinone Oxidoreductase. *J Biol Chem* **263**: 13572–13578

Jernstrom B and Graslund A (1994) Covalent binding of benzo(a)pyrene 7,8-dihydrodiol 9,10-epoxides to DNA: molecular structures, induced mutations and biological consequences. *Biophys Chem* **49**: 185–199

Joseph P and Jaiswal AK (1994) NAD(P)H:quinone oxidoreductase1 (DT diaphorase) specifically prevents the formation of benzo(a)pyrene quinone-DNA adducts generated by cytochrome P4501A1 and P450 reductase. *Proc Natl Acad Sci USA* **91**: 8413–8417

Joseph P, Xie T, Xu YH and Jaiswal AK (1994) NAD(P)H:quinone oxidoreductase 1 (DT-diaphorase): expression, regulation, and role in cancer. *Oncol Res* **6**: 525–532

Kaufman RJ, Davies MV, Wasley LC and Michnick D (1991) Improved vectors for stable expression of foreign genes in mammalian cells by use of the untranslated leader sequence from EMC virus. *Nucl Acids Res* **19**: 4485–4490

Kraemer KH and Seidman MM (1989) Use of *supF*, an *Escherichia coli* tyrosine suppressor tRNA gene, as a mutagenic target in shuttle-vector plasmids. *Mutation Res* **220**: 61–72

Laird PW, Zijderveld A, Linders K, Rudnicki MA, Jaenisch R and Berns A (1991) Simplified mammalian DNA isolation procedure. *Nucleic Acids Res* **19**: 4293

Lind C, Hochstein P and Ernster L (1982) DT-Diaphorase as a quinone reductase: a cellular control device against semiquinone and superoxide radical formation. *Arch Biochem Biophys* **216**: 178–185

Monks TJ, Hanzlik RP, Cohen GM, Ross D and Graham DG (1992). Contemporary issues in toxicology. Quinone chemistry and toxicity. *Toxicol Appl Pharmacol* **112**: 2–16

O'Brien PJ (1991) Molecular mechanisms of quinone cytotoxicity. *Chem-Biol Interact* **80**: 1–41

Paris CN and Seidman MM (1992) A signature element distinguishes sibling and independent mutations in a shuttle vector plasmid. *Gene* **117**: 1–5

Prochaska HJ, Santamaria AB and Talalay P (1992) Rapid detection of inducers of enzymes that protect against carcinogens. *Proc Natl Acad Sci USA* **89**: 2394–2398

Rushmore TH and Pickett CB (1993) Glutathione-S-transferases, structure, regulation and therapeutic implications. *J Biol Chem* **268**: 11475–11478

Sakai A, Miyata N and Takahashi A (1995) Initiating activity of quinones in the two-stage transformation of BALB/3T3 cells. *Carcinogenesis* **16**: 477–481

Sambrook J, Fritsch EF and Maniatis T (1989) Molecular Cloning: a Laboratory Manual. Cold Spring Harbor Laboratory Press: Plainview, NY.

Talalay P, Fahey JW, Holtzclaw WD, Prestera T and Zhang Y (1995) Chemoprevention against cancer by phase 2 enzyme induction. *Toxicol Lett* **82–83**: 173–179

Yamano S, Aoyama T, Mcbride OW, Hardwick JP, Gelboin HV and Gonzalez FJ (1989). Human NADPH-P450 oxidoreductase: complementary DNA cloning, sequence and vaccinia virus-mediated expression and localization of the CYPOR gene to chromosome 7. *Mol Pharm* **35**: 83–88

Zhang Y, Talalay P, Cho C, Posner GH (1992) A major inducer of anticarcinogenic protective enzymes from broccoli: isolation and elucidation of structure. *Proc Natl Acad Sci USA* **89**: 2399–2403

 *© Cancer Research Campaign 1998*